# Carnivorous *Nepenthes x ventrata* plants use a naphthoquinone as phytoanticipin against herbivory

**DOI:** 10.1371/journal.pone.0258235

**Published:** 2021-10-22

**Authors:** Alberto Dávila-Lara, Asifur Rahman-Soad, Michael Reichelt, Axel Mithöfer

**Affiliations:** 1 Research Group Plant Defense Physiology, Max Planck Institute for Chemical Ecology, Jena, Germany; 2 Department of Biochemistry, Max Planck Institute for Chemical Ecology Jena, Germany; Zhejiang University, CHINA

## Abstract

Carnivorous plants feed on animal prey, mainly insects, to get additional nutrients. This carnivorous syndrome is widely investigated and reported. In contrast, reports on herbivores feeding on carnivorous plants and related defenses of the plants under attack are rare. Here, we studied the interaction of a pitcher plant, *Nepenthes x ventrata*, with a generalist lepidopteran herbivore, *Spodoptera littoralis*, using a combination of LC/MS-based chemical analytics, choice and feeding assays. Chemical defenses in *N*. *x ventrata* leaves were analyzed upon *S*. *littoralis* feeding. A naphthoquinone, plumbagin, was identified in *Nepenthes* defense against herbivores and as the compound mainly responsible for the finding that *S*. *littoralis* larvae gained almost no weight when feeding on *Nepenthes* leaves. Plumbagin is constitutively present but further 3-fold increased upon long-term (> 1 day) feeding. Moreover, in parallel *de novo* induced trypsin protease inhibitor (TI) activity was identified. In contrast to TI activity, enhanced plumbagin levels were not phytohormone inducible, not even by defense-related jasmonates although upon herbivory their level increased more than 50-fold in the case of the bioactive jasmonic acid-isoleucine. We conclude that *Nepenthes* is efficiently protected against insect herbivores by naphthoquinones acting as phytoanticipins, which is supported by additional inducible defenses. The regulation of these defenses remains to be investigated.

## Introduction

Carnivorous plants have fascinated people, not only scientists, for much more than 150 years; the time when Charles Darwin’s pioneer studies on this topic was published in his book about ‘Insectivorous Plants’ [1]. In plants, insectivory, or in a broader sense carnivory, has evolved as an additional way to compensate for shortages of soil nutrients such as nitrogen and phosphorus [2, 3]. This feature enables carnivorous plants to grow on nutrient-poor soils. The carnivorous syndrome has evolved independently in angiosperms several times as a result of convergent evolution; thus, carnivorous plants represent a polyphyletic group [4, 5]. Three parameters represent the prerequisites necessary to qualify a plant as carnivorous, i.e. the ability of prey attraction, prey trapping and killing, and prey digestion and nutrients absorption [6]. These prerequisites have been achieved in carnivorous plants by the development of specialized forms, in particular of the trapping mechanisms. Among others, most prominent are the snap-trap of *Dionaea muscipula* (Venus flytrap), the flypaper-traps found in the genera of *Drosera* (sundew) and *Pinguicula* (butterwort), the sucking bladder-traps of *Utricularia spp*. (bladderwort) as well as the pitfall-traps of, for example, *Cephalotus follicularis* and the genera *Sarracenia* and *Nepenthes*, representing new- and old-world pitcher plants. History, systematics and ecology of carnivorous plants has recently been covered by a monograph [7]; newest aspects of molecular evolution and physiology by a review [8].

*Nepenthes* (S1*A* Fig) is a tropical plant genus occurring mainly in Southeast Asia. Due to a slippery surface visiting insect prey falls inside the pitcher traps and drown in a digestive fluid [2]. The genus *Nepenthes* has a large chemical diversity and several secondary metabolites were isolated for pharmaceutical, biotechnological and ethnobotanical use especially in traditional medicine [9, 10]. Many reports describe curative effects of *Nepenthes* extracts on various diseases including hypertension, malaria, and oral cancer [11–16]. Among secondary metabolites, carotenoids, flavonoids, sterols, triterpenes, and naphthoquinones (NQ) are described for *Nepenthes* leaves [2, 17, 18]. In particular NQ are described as antimicrobial metabolites. Interestingly, they are also found in the digestive pitcher fluid, e.g. droserone, 5-*O*-methyl droserone in *N*. *khasiana* [19]; plumbagin, 7-methyl-juglone in *N*. *ventricosa* [20]. Hence, it was hypothesized that these compounds preserve prey during digestion and provide protection against decomposing microbes [19–22]. NQ derivatives are also described for various tissues of *Nepenthes* species including the pitchers [17, 20, 22, 23]. Recently, an untargeted metabolomics approach was performed in *N*. *x ventrata* comparing metabolite features of leaves and pitcher tissue before and after prey captures [24]. About 2,000 compounds (MS/MS events) were detected in the two tissues demonstrating enormous metabolome diversity. Besides a huge number of unknown compounds, the common constituents had an aromatic nature [24]. The tissue metabolite specificity could significantly discriminate leaves and pitchers. This suggests that the metabolite compositions might point to their functions and, furthermore, may represent mechanisms that enable the plants to cope with environmental abiotic and biotic challenges [18].

A typical biotic challenge plants have to deal with is the attack of herbivorous insects. Surprisingly, only few observations and studies are published describing the attack of insects on carnivorous pitcher plants. For instance, lepidopteran herbivory was described for some species of the new world pitcher plant *Sarracenia* [25, 26]. For *Nepenthes*, there is only one investigation showing that *N*. *bicalcarata* plants are infested by the weevil *Alcidodes spec*. [27]. For *N*. *gracilis*, it has been shown that green pitchers experience more herbivory than red ones [28]. To the best of our knowledge, up to now no other studies have been published that focus on herbivore damage in *Nepenthes*. Seemingly, herbivory on *Nepenthes* tissue is rare. The reason behind is not known yet. However, it is unlikely that all putative herbivores are caught and digested; more likely, *Nepenthes* is equipped with an efficient setting of defensive chemistry, as known for numerous plants [29]. For example, the presence of jasmonates and the related signaling pathway involved in the induction of plant defenses against herbivory is present in carnivorous plants such as *Drosera capensis*; *Dionaea muscipula; Nepenthes spp*. as recently reviewed [30]. Moreover, very recently it has been shown that the *Nepenthes* tissue-derived NQ plumbagin has anti-feeding and insect growth-inhibiting properties [23]. However, no *in vivo* feeding experiments on intact *Nepenthes* plants have been done yet and whether or not the NQ plumbagin is a key player in *Nepenthes*’ defense remained an open question. Nevertheless, also in *Nepenthes* chemical defense against feeding insects is most likely established and, maybe, this defense is highly efficient.

In this study, we addressed these issues to learn how a carnivorous *Nepenthes* plant responds to insect attack. We performed choice and feeding experiments employing larvae of the generalist herbivorous moth *Spodoptera littoralis* and *Nepenthes x ventrata* as host plant to investigate the insect’s behaviour and growth performance. Feeding-induced quantitative changes in phytohormone and defensive plumbagin levels were analyzed in *N*. *x ventrata* leaf blades using LC/MS. We further examined the possibility of a phytohormone dependent induction of plumbagin accumulation and other defense-related compounds. Our data suggest that in *Nepenthes* besides prey digestion the jasmonate signaling pathway is also involved in defense induction but increase of constitutive defense is jasmonate independent.

## Material and methods

### Plants and insects

*Nepenthes x ventrata* Hort. ex Fleming plants, natural hybrids of *N*. *alata* Blanco *x N*. *ventricosa* Blanco, were bought from a company (Gartenbau Carow, Nürtlingen, Germany) about 20 year ago and ever since grown in the MPI greenhouse on peat substrate at 23–25°C, 80–100% relative humidity, and a 16/8 h light/dark photoperiod. All plants were at least 2 years old. Plant pots were shuffled once in every three months to ensure optimum space and equal support for growth and to avoid any edge effect, caused by differences in micro environmental conditions between the core and at the edge of the samples group. *N*. *x ventrata* leaves used for the experiments had fully expanded but only contained pitcher buds to focus on the leaf chemistry only without having the influence of the mature pitcher chemistry.

Larvae of the insect *Spodoptera littoralis* Boisd. (Lepidoptera: Noctuidae) were hatched from eggs (provided by Syngenta Crop Protection, Stein, Switzerland) and reared on artificial diet as described [23] at 23–25°C with a 14/10 h light/dark photoperiod. As a generalist, this moth is frequently used in feeding experiments. It is closely related to *S*. *litura*, a species that occurs in the same regions as *Nepenthes*.

### Feeding and choice assays

#### Feeding assays

Here, second to third instar *S*. *littoralis* larvae were used. To avoid cannibalism, all feeding assays were performed with individual larvae. Assays with artificial diet supplemented with plant tissue or plumbagin (5-hydroxy-2-methyl-1,4-naphthoquinone, C_11_H_8_O_3_; Fischer Scientific, Schwerte, Germany) were done as described [23]. The larvae were kept in a controlled temperature cabinet at 25 ± 1°C and 60–70% relative humidity. Every day fresh diet was provided. For these feeding assays, 15 independent repeats were done.

To analyze weight gain of *S*. *littoralis* feeding on a *N*. *x ventrata* leaf, individual 3rd instar larvae were used. The larvae were left to feed on the same leaf for five days in the greenhouse. The whole leaf was covered with an air- and water-permeable polyethylene terephthalate (PET) bag (Toppits, Minden, Germany) to prevent escaping of the larvae and keep them on the particular leaf (S1*B* Fig). Larvae were carefully recovered and re-weighed every day. The number of dead individuals was recorded. For control, larvae growing on artificial diet were placed for the same time and under the same conditions near to the *N*. *x ventrata* plants.

#### Feeding-induced defense

For short term herbivory, leaves were infested with a single 3rd instar *S*. *littoralis* larvae starved for 24 h before starting the experiment. The caterpillar was placed carefully on the *N*. *ventrata* leaf with a tweezer. Lightweight cage (PET; 10 cm diameter, 1 cm height) was placed on the leaf covering both sides to prevent escaping of the larvae. For long-term herbivory, PET bags were used instead of cages to cover the entire leaf. All herbivory experiments were performed in the greenhouse.

#### Choice assay

To measure the preference of larvae towards *N*. *x ventrata* leaf or pitcher tissue, 15 larvae were confined in a square (12 x 12 cm) petri dish containing a single piece (*c*. 10 cm^2^) of leaf and pitcher tissue each in a choice situation. The bioassay was conducted for fixed time period and the choice made by the larvae for a specific tissue was assessed after 30, 60, and 180 min

### Phytohormone treatment

For the phytohormone spraying experiment, each treatment group contained five individual plants on a single tray. Trays were placed with sufficient distance to avoid drift of the spray to other treatment groups. Plants were foliar-sprayed (4 mL plant^-1^) twice, at the beginning and after 12 h only with one of the phytohormones jasmonic acid (JA, 500 μM), abscisic acid (ABA, 250 μM), salicylic acid (SA, 250 μM) (all from Sigma-Aldrich, Taufkirchen, Germany) for each treatment group. Spraying solutions were made from 50 mM (JA), and 100 mM (ABA, SA) stock in absolute ethanol and the final volume was adjusted to 20 mL with ddH_2_O for each. While spraying, already opened pitcher traps were shaded to avoid droplets entering inside traps. For the control group under the same conditions, plants were sprayed with the same solution without any phytohormone. Leaves were harvested 24 h after first spraying, directly frozen in liquid nitrogen and stored at -80°C for further analysis.

### Phytohormone and plumbagin extraction and quantification

Sampled *N*. *x ventrata* leaves were finely-ground in liquid nitrogen using mortar and pestle. Approximately 100 mg of finely ground leaf tissue was weighed in 2 mL tubes. The extraction, detection and quantification of phytohormones were performed as previously described [31] with modifications [32] using an LC-MS/MS system. For analysis, each sample was extracted with 1.5 mL methanol containing 60 ng D_6_–abscisic acid (Toronto Research Chemicals, Toronto, Canada), 60 ng of D_6_–jasmonic acid (HPC Standards GmbH, Cunnersdorf, Germany), 60 ng D_4_–salicylic acid (Santa Cruz Biotechnology, Santa Cruz, U.S.A) and 12 ng of D_6_-jasmonic acid-isoleucine conjugate (HPC Standards GmbH, Cunnersdorf, Germany) as an internal standard.

Phytohormone and plumbagin analyses were combined and performed by LC-MS/MS as described [31, 32] on an Agilent 1260 series HPLC system (Agilent Technologies, Böblingen, Gemany) with the modification that a tandem mass spectrometer QTRAP 6500 (SCIEX, Darmstadt, Germany) was used. Briefly, chromatographic separation was achieved on a Zorbax Eclipse XDB-C18 column (50 x 4.6 mm, 1.8 μm, Agilent Technologies). Water containing 0.05% formic acid and acetonitrile were employed as mobile phases A and B, respectively. The elution profile was: 0–0.5 min, 10% B; 0.5–4.0 min, 10–90% B; 4.0–4.02 min, 90–100% B; 4.02–4.5 min, 100% B and 4.51–7.0, min 10% B. Flow rate was kept at 1.1 mL min^-1^ and the column temperature was maintained at 25°C. The mass spectrometer was equipped with a Turbo spray ion source operated in negative ionization mode. The ion spray voltage was maintained at -4,500 eV. The turbo gas temperature was set at 650°C. Nebulizing gas was set at 60 psi, curtain gas at 40 psi, heating gas at 60 psi, and collision gas was set to “medium”. The mass spectrometer was operated in multiple reactions monitoring (MRM) mode, details of the instrument parameters and response factors for quantification can be found in S1 Table. For plumbagin an MRM was added to the method: Q1: m/z 187, Q3: m/z 159, DP: -20, CE: -18. Since we observed that both, the D_6_-labeled JA and D_6_-labeled JA-Ile standards (HPC Standards GmbH, Cunnersdorf, Germany) contained 40% of the corresponding D_5_-labeled compounds, the sum of the peak areas of D_5_- and D_6_-compound was used for quantification. For quantification of plumbagin, the internal D_6_-JA standard was used applying an experimentally-determined response factor of 164. The response factor was determined by analyzing a mixture of plumbagin (insert supplier of standard here) and D_6_-JA at the same concentration.

In addition to using LC-MS/MS for plumbagin quantification, a second method using HPLC-UV was also applied using the same extraction protocol. A 20 μL aliquot of the methanolic extract was separated using high performance liquid chromatography (Agilent 1100 HPLC system, Agilent Technologies) on a reversed-phase C-18 column (Nucleodur Sphinx RP, 250 x 4.6 mm, 5 μm, Macherey-Nagel, Düren, Germany) with a 0.2% formic acid in water (A)-acetonitrile (B) gradient (0 min, 20% B; 0–14 min, 20–76% B; 14–14.1 min, 76–100% B; 14.1-16min 100% B and 16.1–20 min 20% B; flow rate 1.0 mL min^-1^). Detection was performed with a photodiode array detector, and peaks were integrated at 265 nm. Quantification of plumbagin (Fischer Scientific, Schwerte, Germany) was achieved by generating a plumbagin standard curve in the range of 8 to 250 μg mL^-1^.

### Protease inhibitor assay

*Nepenthes x ventrata* protein was extracted following the Pierce Plant Total Protein Extraction Kit (ThermoFischer Scientific, Darmstadt, Germany) protocol with minor modifications. For extraction, the native lysis buffer was diluted (1:1) with 50 mM Tris-HCl, pH 7.4 containing 30 mM CaCl_2_ and 2 mL Eppendorf tubes were used. For each sample, 50 mg freshly ground tissue was mixed with 250 μL extraction buffer. The homogenate was shortly vortexed, and then incubated on ice for 5 min following a centrifugation step for five min at 16,000 x *g* and at 4°C. The supernatant was recovered for further analysis, i.e. 150 μL extract was recovered and used for protein quantification and trypsin protease inhibitory (TI) activity measurements. The TI’s activity was assayed by determining the residual trypsin activity according to [33] using BApNA (Nα-Benzoyl-L-arginine 4-nitroanilide hydrochloride; Sigma-Aldrich) as substrate and bovine trypsin (Sigma-Aldrich) as the standard enzyme. The reaction mixture containing 50 μL leaf protein extract, 20 μL trypsin (1 mg mL^-1^ in 50 mM Tris-HCl, pH 7.4 containing 30 mM CaCl_2_) was incubated at 37°C for 15 min with shaking (700 rpm). Then, 40 μL of BApNA (10 mg mL^-1^ in DMSO) was added to the assay solution and the reaction mixture was incubated at 37°C for 20 min with shaking (900 rpm) followed by termination of the reaction by adding 500 μL of 10% (v/v) glacial acetic acid. A blank for each sample was run simultaneously to subtract the absorbance caused by the extract. In blank, acetic acid was added before BApNA. For a positive control, only extraction buffer was added instead of sample extract to avoid any inhibition, representing maximum trypsin activity. The absorbance was recorded at 410 nm alongside the blank with a spectrophotometer.

### Statistical analyses

Statistical calculations were performed using GraphPad Prism version 9.0.0 in all cases. Details are indicated in the particular figure legends. For growth experiments, larvae were picked randomly from a large population and all experiments were conducted out under highly standardized conditions to avoid investigator-included bias.

## Results

### Plumbagin in artificial diet and *Spodoptera littoralis* larva growth

**Fig 1:**
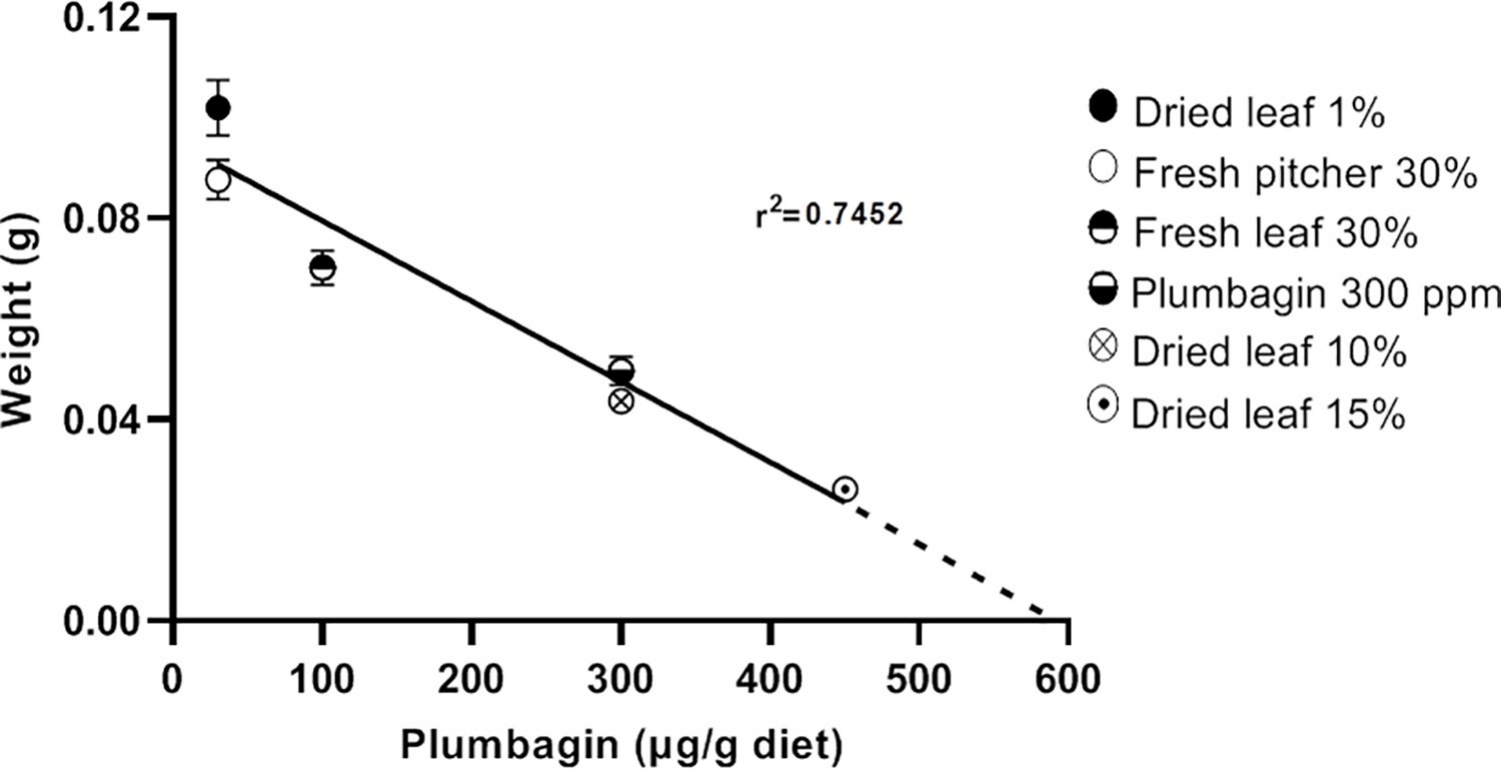
*Spodoptera littoralis* larvae growth *vs*. different plumbagin concentrations in artificial diet. Plumbagin from various sources (represented by different symbols) was included in the diet. In all assays 2nd instar larvae were used; weight was determined at day 5. Regression showed statistical significance with *p* < 0.0001 (estimated r^2^ = 0.745, F = 251.5 (DFn = 1, DFd = 86), alpha level = 0.05). The r^2^ value (0.745) indicates a strong relationship between larval development and plumbagin concentrations present in the diet.

Recently published data on *S*. *littoralis* larvae feeding on artificial diets that was supplemented with different *Nepenthes* tissue showed that the larvae gained less weight the more tissue was added [23]. We re-analyzed those data (fresh pitcher, 30%; fresh leaf, 30%; dried leaf, 10%, 15%) and calculated the amount of plumbagin that was supplemented with the particular tissue. Two more data sets were added (dried leaf, 1%; plumbagin). Based on these data it can be seen that the increase of plumbagin concentration in the diet negatively correlates with weight gain of the larvae and this effect was independent of the plumbagin source (Fig 1).

### *Spodoptera littoralis* food choice assay

**Fig 2:**
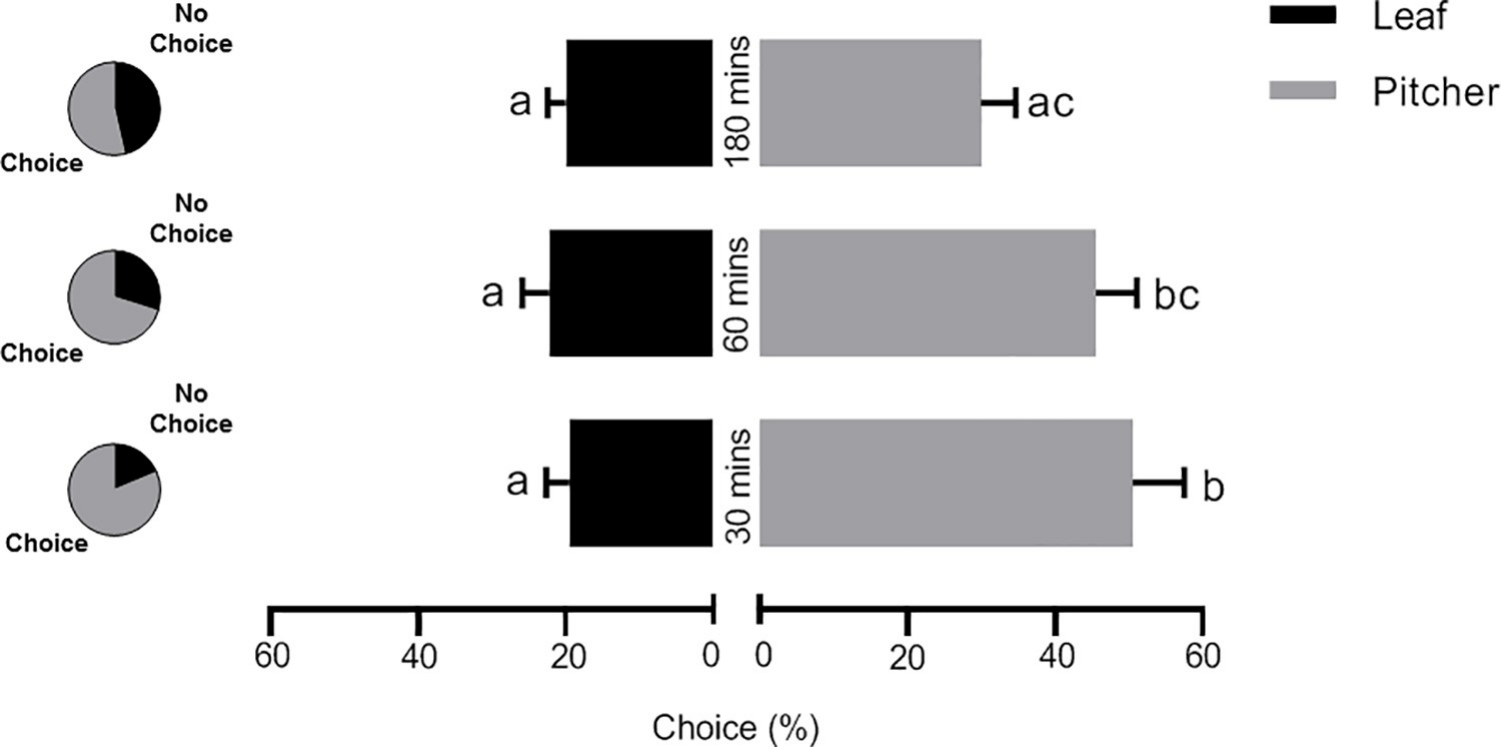
*Spodoptera littoralis* larvae food choice assay for *Nepenthes x ventrata* pitcher and leaf tissue. The proportions of larvae (15 per experiment) making choices are indicated in pie charts. Mean (± SEM), n = 12; two-way ANOVA, Šidák’s multiple comparisons test; different letters at the end of each bar indicate significant difference (*p* < 0.05).

Knowing that the plumbagin concentration in leaf tissue is about 5-times higher than in pitcher tissue and larvae feeding on diet supplemented with pitcher tissue gain more weight [23] (Fig 1), a choice assay was conducted to see if the larvae prefer to feed on leaf or pitcher tissue. Fifteen *S*. *littoralis* larvae were placed in a petri dish with an equal piece each of *N*. *x ventrata* leaf and pitcher tissue. After 30, 60, and 180 min the number of larvae feeding on either tissue was noted. As shown in Fig 2, the larvae had a clear and significant preference for the pitcher tissue at all time points. While over the whole period only *ca* 20% of the larvae chose to stay close to or on the leaf, around 50% chose the pitcher after 30 and 60 min. Only after 180 min the number dropped below 40%. Over time also the number of larvae that decided for either tissue dropped. Strikingly, only the pitcher tissue was eaten. The ongoing feeding process was documented to see the remained size of the tissues (S2 Fig).

### *Spodoptera littoralis* feeding on *Nepenthes x ventrata* leaves

**Fig 3:**
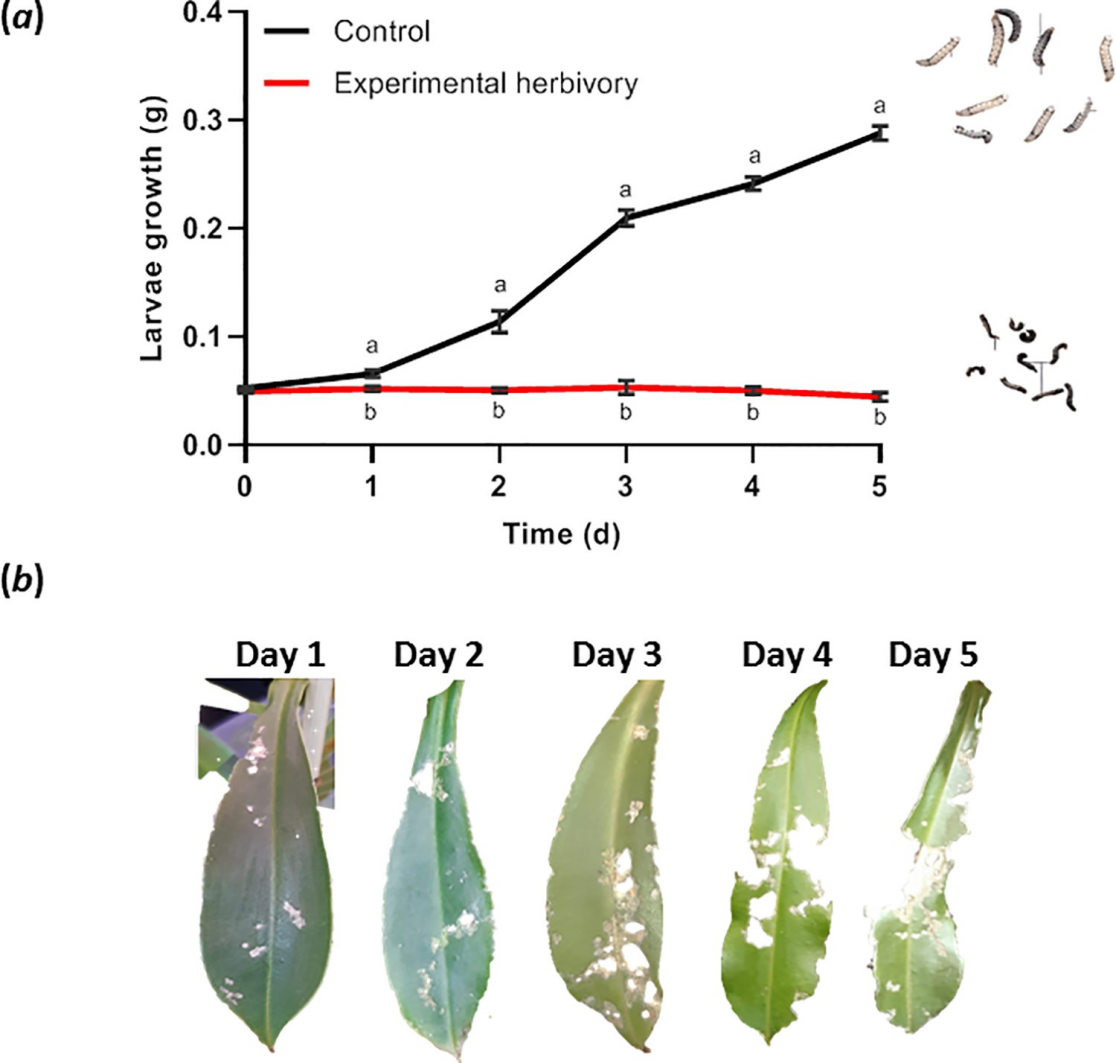
Performance of *Spodoptera littoralis* larvae feeding on *Nepenthes x ventrata* leaves. (a) Change of larvae weight when feeding on leaves (experimental herbivory) or artificial diet (control). Larvae were weighed every day for 5 days. Mean (± SEM), *n* = 15; two-way ANOVA, Šidák’s multiple comparisons test; different letters indicate significant difference (*p* < 0.05). Photos of larvae were taken at day 5, squares are 1 cm^2^. (*b*) *N*. *x ventrata* leaf eaten; photos taken at days 1 to 5.

Next, we wanted to study the effects of *N*. *x ventrata* leaves on the feeding behavior of *S*. *littoralis* larvae when there is no alternative food. Therefore, the larvae were placed on the leaves without the chance to escape. As control, artificial diet was offered close to the plants under the same environmental conditions. While in the latter case larvae showed normal feeding behavior and weight increase, the larvae that fed on the leaves did not gain weight at all, not even after 5 d (Fig 3*A*). This was not due to feeding avoidance as the leaves were eaten and the leaf area was continuously reduced over time (Fig 3*B*). Moreover, if the larvae were taken after 5 d and put on artificial diet, they started growing and developed as normal.

### Herbivory and plumbagin accumulation

**Fig 4:**
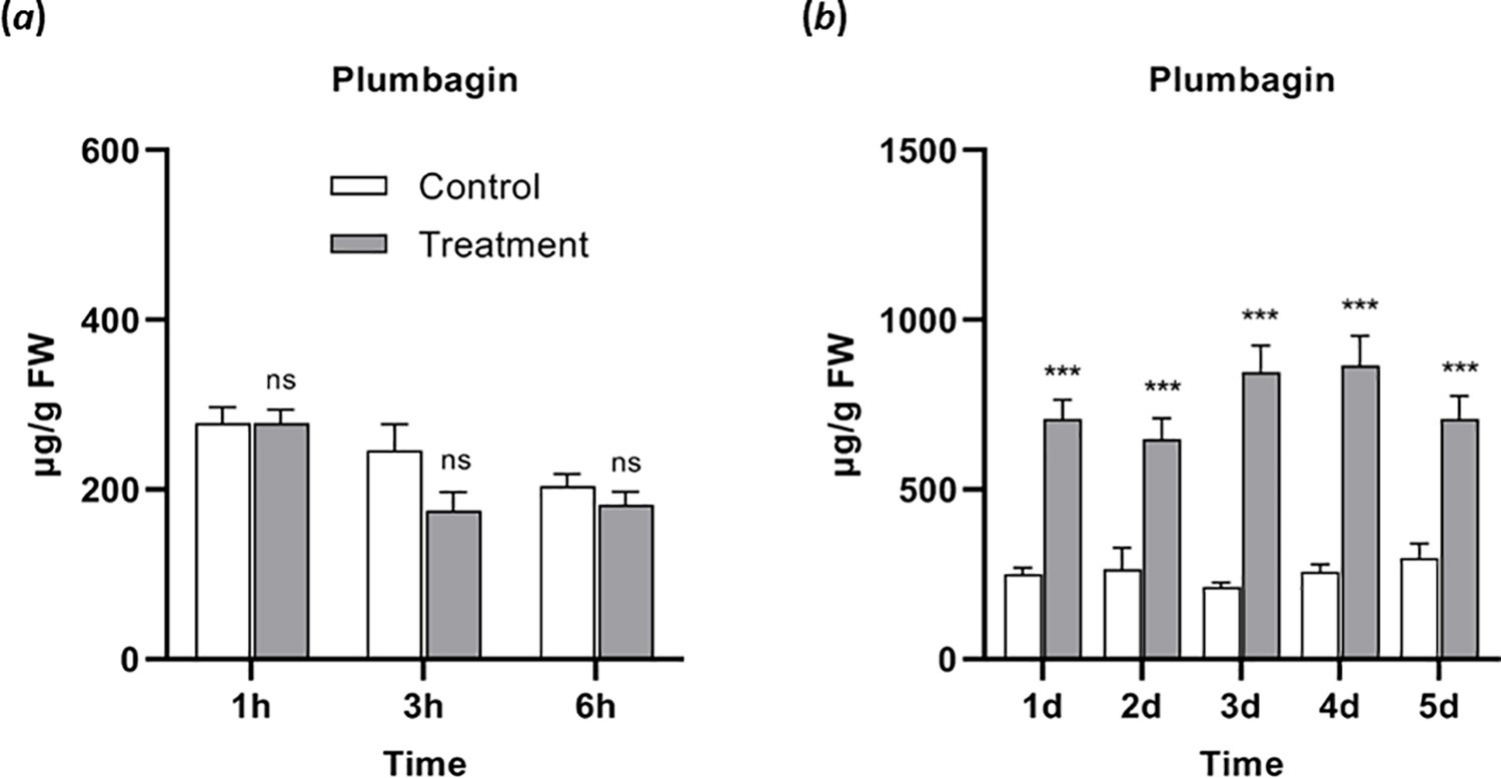
Plumbagin accumulation in *Nepenthes x ventrata* leaves upon *Spodoptera littoralis* larvae feeding. (*a*) Plumbagin content after short term feeding. (*b*) Plumbagin content after long term feeding. Mean (± SEM), *n* = 10; unpaired t-test with Welch correction; ns = not significant; *** *p* < 0.001.

To further investigate the role of plumbagin in this process, the plumbagin levels were analyzed over time. A constitutive level of this NQ (256 ± 13.5 μg g^-1^ fresh weight; n = 25; mean ± SEM) was detected in the leaves. To examine any additional effect of *S*. *littoralis* herbivory, both short- and long-term accumulation of plumbagin was determined. Within the first 6 h of feeding, no significant change of plumbagin level was detected in the leaves (Fig 4*A*); however, after day 1 to day 5, a *ca*. 3-fold increase of plumbagin was determined (755 ± 43.0 μg g^-1^ fresh weight; n = 50; mean ± SEM) that stayed at this level during the feeding process (Fig 4B).

### Herbivory-changed phytohormone level in *Nepenthes x ventrata* leaves

**Fig 5:**
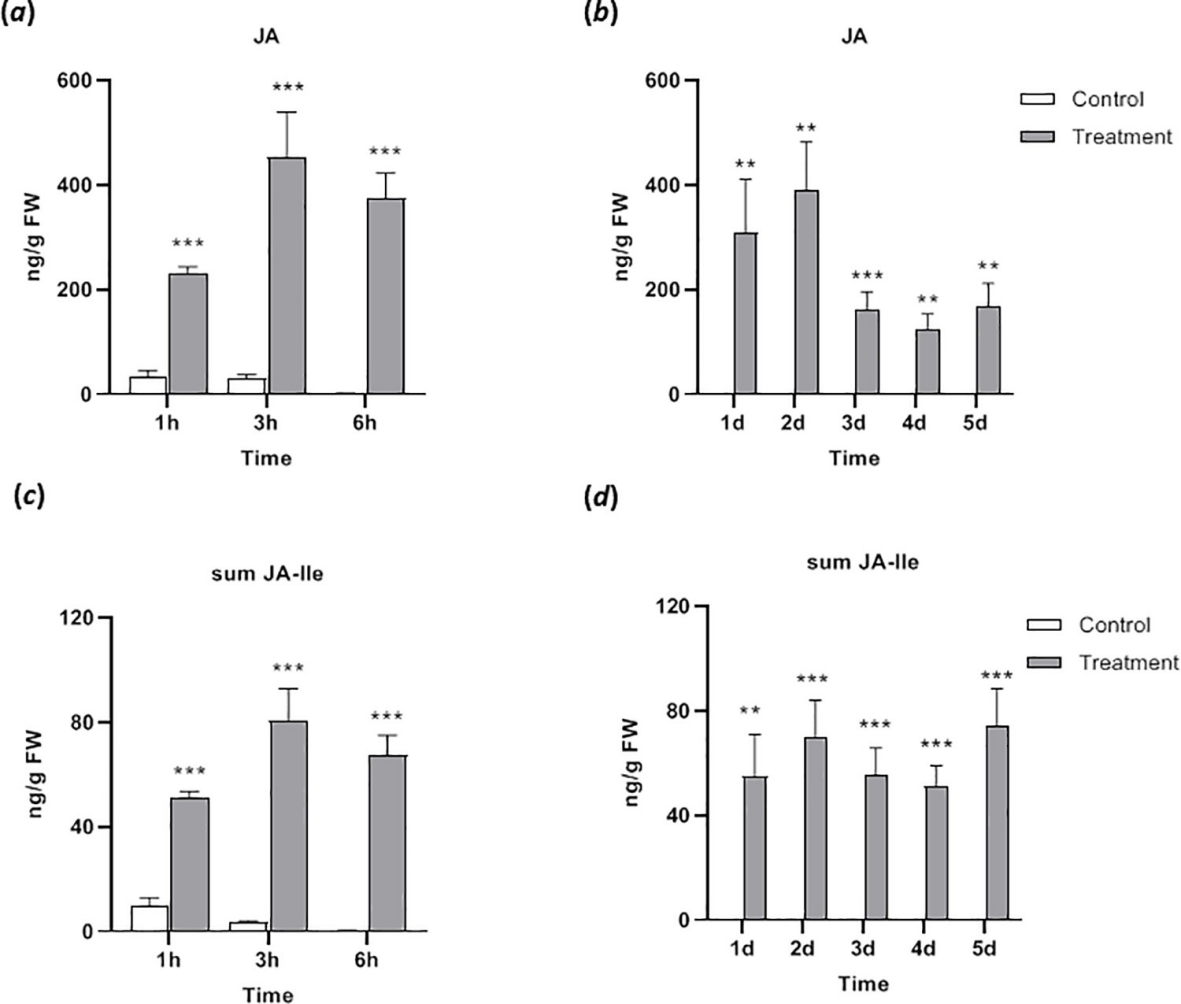
Jasmonates in *Nepenthes x ventrata* leaves upon *Spodoptera littoralis* larvae feeding. (*a*) Jasmonic acid (JA) content after short term and (*b*) long term feeding. (*c*) Jasmonic acid-isoleucine conjugate (JA-Ile) content after short term and (*d*) long term feeding. Mean (± SEM), *n* = 10; unpaired t-test with Welch correction; *** *p* < 0.001; ** *p* < 0.01.

Because herbivore feeding typically induces an increase of certain phytohormones, in particular of jasmonates, in the infested tissues, various phytohormones were analyzed at the same time points as we analyzed plumbagin. A significant fast and drastic increase of both JA and JA-Ile was found after 1 h, staying high during the short term analyses (Fig 5*A* and 5*C*). In the long-term analyses, the JA level was kept high until day 2 and dropped by half after day 3 to day 5; nevertheless, these JA levels were still significantly higher than in the controls (Fig 5*B*). In case of JA-Ile, the same level (around 60 ng g^-1^ fresh weight) was kept for the 5 d period of the whole feeding experiment (Fig 5*D*). Degradation products of JA-Ile, the bioactive jasmonate, were examined as well. Again, a fast increase of their accumulation was found after 1 h for OH-JA-Ile, which stayed more or less at the same level until day 5 (S3*A* and S3*B* Fig). For COOH-JA-Ile the situation was slightly different. Only after 3 h a significant increase was found and a second 2-fold increase could be detected at day 2 (S3*C* and S3*D* Fig).

**Fig 6:**
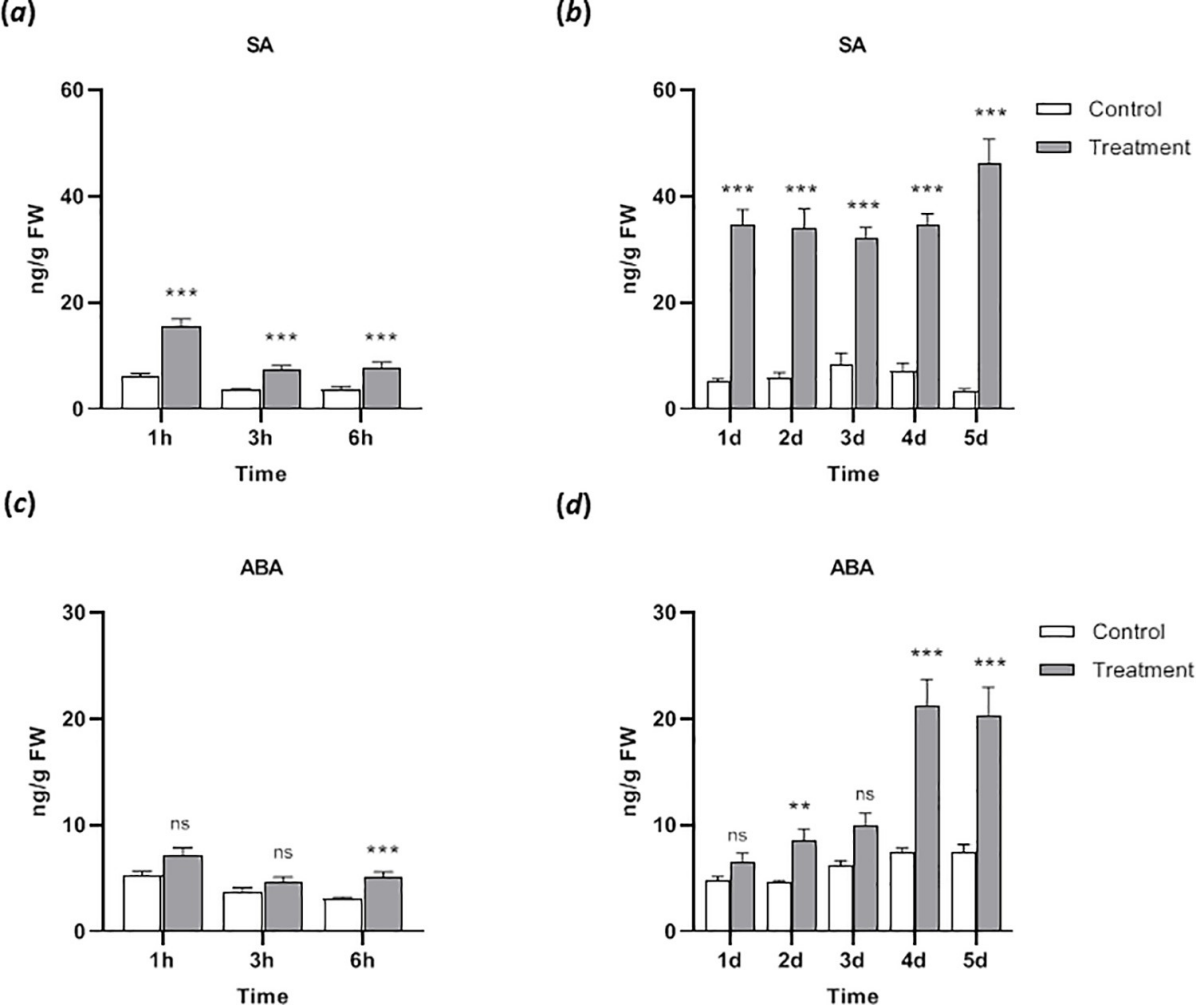
Salicylic acid (SA) and abscisic acid (ABA) in *Nepenthes x ventrata* leaves upon *Spodoptera littoralis* larvae feeding. (*a*) SA content after short term and (*b*) long term feeding. (*c*) ABA content after short term and (*d*) long term feeding. Mean (± SEM), *n* = 10; unpaired t-test with Welch correction; ns = not significant; *** *p* < 0.001; ** *p* < 0.01.

Besides jasmonates, the phytohormones SA and ABA were investigated. For SA a slight but significant 2-fold increase was found in the short term analysis that further increased at day 1 (around 5-fold) and stayed at that level for the whole 5 d period (Fig 6*A* and 6*B*). In case of ABA only after 6 h a weak tendency for higher ABA content was recognized that continuously increased and eventually showed a clear accumulation at days 4 and 5 (Fig 6*C* and 6*D*).

### Phytohormone-mediated accumulation of plumbagin

**Fig 7:**
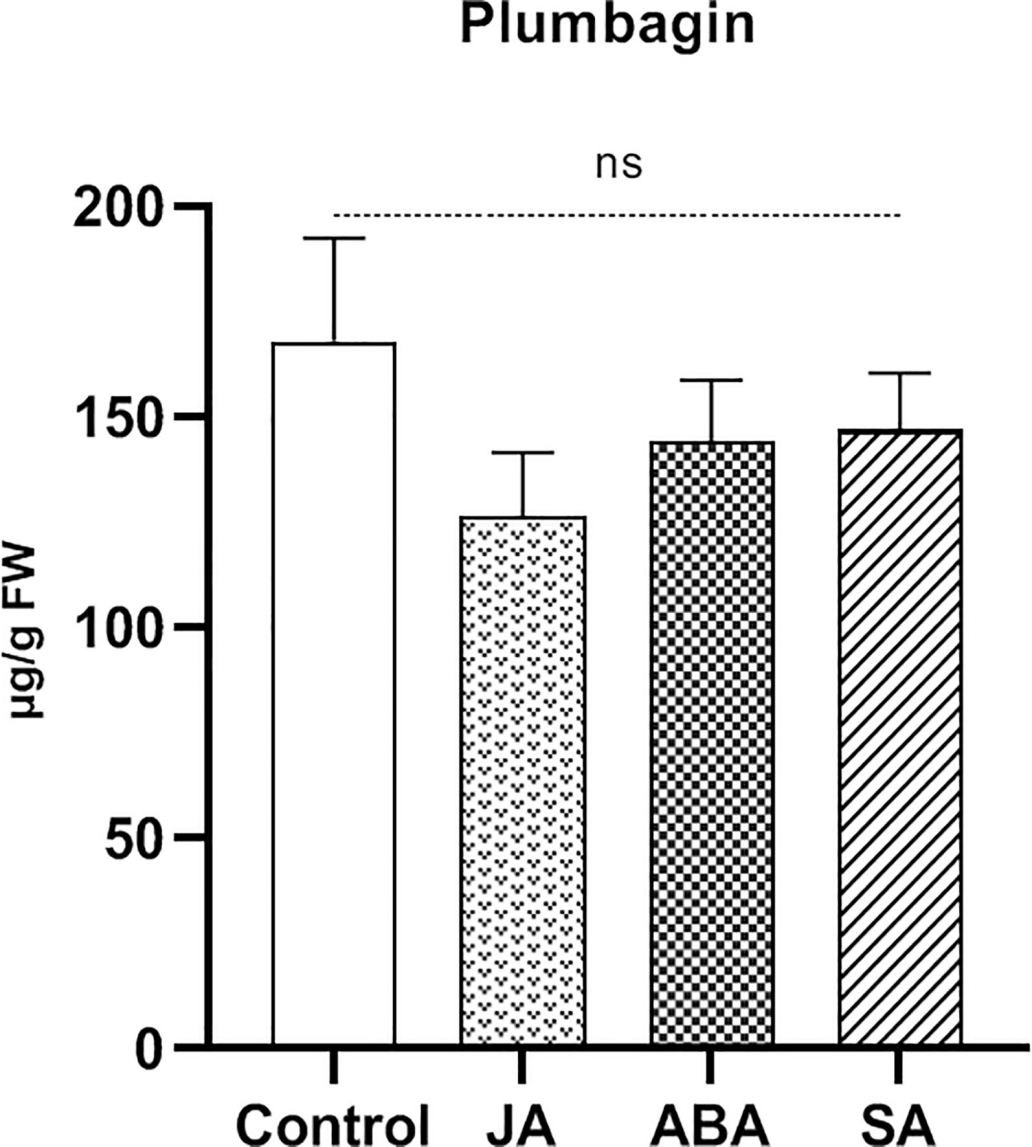
Phytohormone-induced plumbagin accumulation in *Nepenthes x ventrata* leaves. Foliar spray with JA (500 μM), ABA (250 μM), or SA (250 μM) was performed at the beginning (2 mL) and after 12 h (2 mL). Plants were harvested after 24 h. Mean (±SEM), n = 5; one-way ANOVA followed by Tukey’s multiple comparisons test; different letters indicate significant difference *(p* < 0.05); ns = not significant.

In order to find out whether or not any phytohormone may be involved in the induction of plumbagin accumulation during long-term feeding, leaves of *N*. *x ventrata* were treated with phytohormones and plumbagin contents were determined after 24 h. Therefore, solutions of JA, ABA and SA in water were exogenously applied to the leaves of individual plants. No significant difference in the endogenous level of plumbagin was observed in either treatment compared to control or between the treatment groups after 24 h post foliar spray (Fig 7).

### Herbivory- and phytohormone-induced protease-inhibitor activity

**Fig 8:**
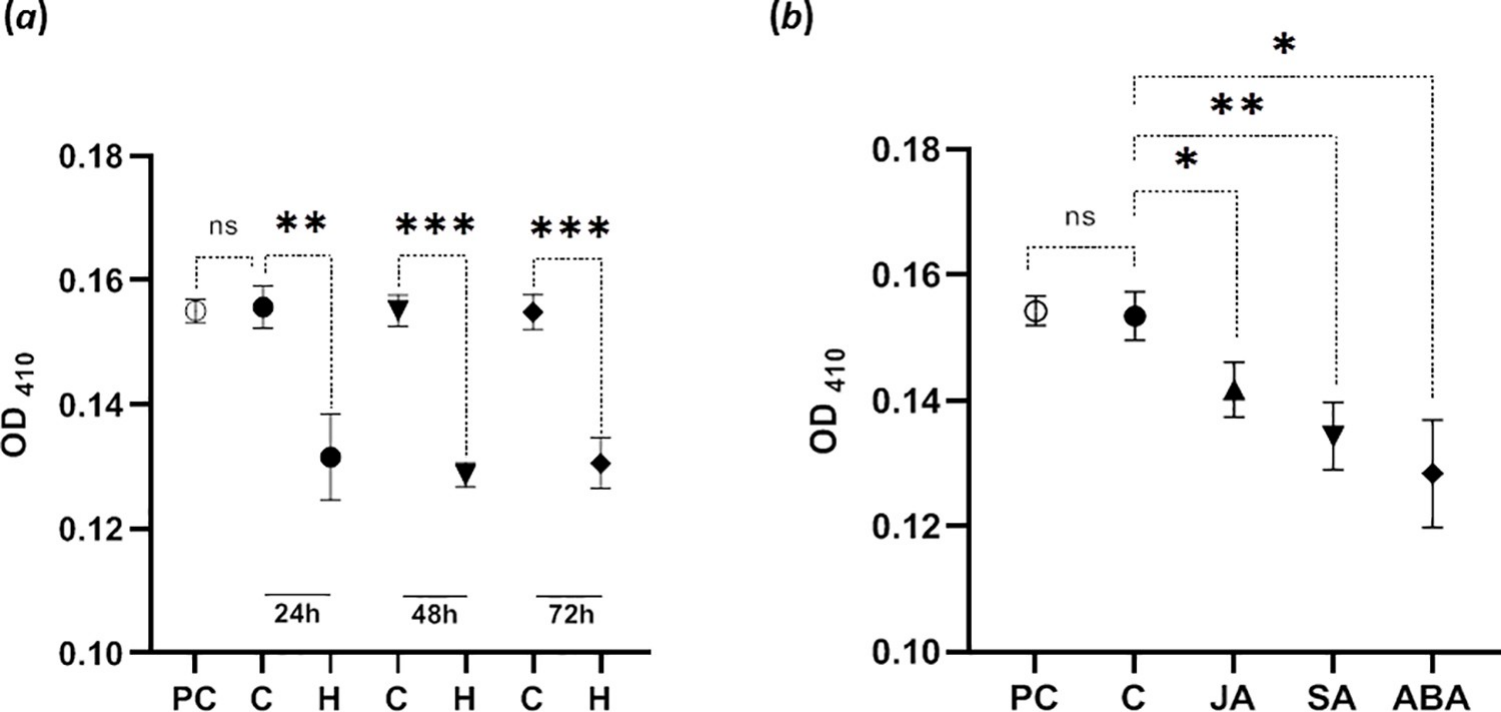
Trypsin protease inhibitor (TI) activities in *Nepenthes x ventrata* leaves. (*a*) TI activities determined after *Spodoptera littoralis* larvae feeding (H) for 24 h, 48 h, and 72 h compared to the respective controls (PC, positive control; C, treatment control). (*b*) TI activities determined after foliar spray with JA (500 μM), ABA (250 μM), or SA (250 μM). Treatment was performed at the beginning (2 mL) and after 12 h (2 mL). Plants were harvested after 24 h. Mean (±SEM), n = 5; unpaired t-test with Welch’s correction; asterisks represent difference between individual treatments vs control; ns = not-significant, * p < 0.05, ** p ≤ 0.01; *** p < 0.001.

Besides plumbagin, very likely other defense component are present in *Nepenthes* leaves that might affect the growth of feeding larvae. Thus, we studied the presence of protease (trypsin) inhibitor (TI) activity as example for a wide spread defense mechanism that could also affect the larvae feeding. The TI activity was assayed after 24 h, 48 h and 72 h of *N*. *x ventrata* leaf infestation by *S*. *littoralis* larvae. The results showed a significant increase of TI activity in herbivore infested leaves in all treatment groups compared to non-infested leaves as controls at all time points (Fig 8*A*). All OD_410_ values from control groups showed a similar range at all times. This, together with the positive control value suggests the absence of trypsin protease inhibitory activity from protein extracts of undamaged leaves alongside excluding the possibility of the involvement of other metabolites from leaf extract causing trypsin inhibition during the assay. The same samples used for foliar spray experiments to look for plumbagin induction (Fig 7) were analyzed for TI activities as well. Statistically significant differences were observed for all phytohormone-treated samples at 24 h after foliar spray compared to the untreated control groups (Fig 8*B*).

## Discussion

While most of the phytochemical studies done on *Nepenthes* and other carnivorous plants mainly addressed carnivorous syndrome-related processes, demonstration of secondary metabolite synthesis and accumulation as a result of herbivory is surprisingly limited. The present study illustrates a defense response in *N*. *x ventrata* against insect herbivory that is based on NQ, in particular on plumbagin. The presence of NQ has been described for many carnivorous plants [34] belonging to the order Nepenthales [18], a *sensu stricto* sister group to Caryophyllales [35] and including the plant families Droseraceae and Nepenthaceae. This includes species such as *Aldrovanda vesiculosa*, *Dionaea muscipula*, *Drosophyllum lusitanicum*, and *Triphyophyllum peltatum* as well as the genera *Drosera* and *Nepenthes* [34].

The function of plant secondary metabolites often lays in their ecological significance in defense against pathogens and herbivores. The observation that *Nepenthes* plants are rarely infested by insect herbivores could be an example of such phenomenon knowing that NQ are bioactive compounds with insect anti-feeding activity [23]. In that study, plumbagin was found as a dominant secondary metabolite in the tissue of *N*. *x ventrata* and its function was evaluated by means of the interaction with a generalist insect herbivore, *S*. *littoralis*.

Experiments with artificial diet containing plumbagin from different sources showed an adverse effect on the growth of the *S*. *littoralis* larvae due to increasing plumbagin concentrations (Fig 1). This negative effect of high plumbagin concentration on feeding larvae was supported in a choice assay where larvae could choose between *N*. *x ventrata* leaf and pitcher tissue. Here, a clear preference for pitchers was found independent on exposure time (Fig 2). This corresponds well to the *ca*. 5-fold lower plumbagin content in pitcher tissue [23]. However, artificial diet sometimes comes with the limitation of being suboptimal or super optimal [36]. Compared to most susceptible host plants, artificial diet often fosters more rapid growth [37] thus making insects less susceptible to the tested compound or extract incorporated in diet. This situation was eliminated by letting the larvae feed directly on *N*. *x ventrata* leaf tissue. Moreover, in a long-term feeding assay on *N*. *x ventrata* leaves, *S*. *littoralis* larvae showed no weight gain at all although they fed a lot (Fig 3). For other lepidopteran species such as *S*. *litura*, *Achaea janata*, and *Trichoplusia ni*, the effect of plumbagin on feeding behavior has also been reported [38–40]. However, the focus of those studies was on the level of feeding avoidance rather than on larval growth and development.

Nevertheless, the finding that *Nepenthes* leaves contain more plumbagin than pitchers and that the latter were preferred by *S*. *littoralis* in selection tests is somewhat reminiscent of the so-called push-pull strategy in agriculture. In this strategy, a repellent (push) and an attractive (pull) resource are combined to push pests away from crops and attract them elsewhere [41]. It is conceivable that *Nepenthes* takes the same approach, pushing insects away from leaves toward pitcher traps where plumbagin concentrations are lower and an attractive cue awaits the extrafloral nectar [42].

Herbivory causes a series of different reactions in the infested plant. The feeding process leads to the rapid induction of signals including phytohormone (mainly jasmonates) accumulation and finally the activation of wound and herbivory-related defenses [29, 43]. Interestingly, jasmonate signaling is also present in carnivorous plants, as demonstrated for the regulation of the digestive process [30]; for example, in *Dionaea muscipula* [44–47], *Drosera capensis* [48], and *Nepenthes alata* [49]. In addition, in *Drosera capensis* the accumulation of jasmonate upon wounding and treatment with insect-derived oral secretion has been shown [50]. The current study revealed the same jasmonates are also involved in the response of *N*. *x ventrata* upon *S*. *littoralis* herbivory. The feeding-stimulated elevation of JA and its biologically active form JA-Ile was rapid and significantly enhanced within the first hour of treatment (Fig 5). The timing of the jasmonate response is comparable with the situation in other herbivore-infested plants; however, there is a clear discrepancy to the slow accumulation of jasmonates upon prey capture in pitcher tissue of *Nepenthes* that was detectable only after 24 hours [49]. At first glance, the different kinetics of jasmonate accumulation in defense and carnivory suggests a completely different regulation of both processes. However, it might be that the initial signal perception and signaling process leading to jasmonate induction is equally fast but the release of a signal (e.g. chitin) and its presentation might be much slower due to a previously necessary digestive process.

To the best of our knowledge, in almost no previous study done on *Nepenthes*, the role of phytohormones has been extensively investigated outside the carnivorous syndrome context. Only studies on the prey-induced responses in *N*. *alata* from Yilamujiang et al. [49] found doubling of endogenous SA level after prolonged (48 h) chitin treatment compared to the original level in the pitcher tissue. Slightly similar but again a faster situation was observed in our current study where in short-term herbivory endogenous level of SA nearly doubled at all time points; after 24 hours the SA level further increased and was found to be stable for the next four days (Fig 6*A* and 6*B*). Typically, SA concentrations increase in response to pathogen infection but not as a general wound response [51, 52]. Therefore, one possibility of increased concentration of SA observed upon prolonged herbivory in this study could be a consequence of increased vulnerability of leaves after herbivore damage that may create open passages for pathogens to enter. Considering stress-related hormones such as ABA [53], this study showed elevated endogenous ABA levels upon long term herbivory (Fig 6*D*); a common response known in plants after infestation by natural enemies [54]. However, increases in ABA are not specific to processes that are associated with induction of resistance; many other conditions such as water stress also cause ABA accumulation but do not produce other manifestations of induced resistance. In this investigation long-term herbivory of leaf tissue caused early senescence, which consequently increases dryness of wounded leaves; this is a possible reason for ABA accumulation over time.

A striking result was the finding that larvae feeding on *N*. *x ventrata* leaves gained almost no weight at all (Fig 3*B*), in contrast to the larvae feeding on enriched diets. This suggested that besides plumbagin an additional factor in the *Nepenthes* tissue might have contributed to this effect. Thus, apart from plumbagin accumulation in *N*. *x ventrata* as a mode of defense response against herbivores, the presence of other factors might be present, which may affect food digestion in the larvae and cause less or no weight increase. Such a factor and known defensive tools are protease inhibitors [29]. As demonstrated in Fig 8*A*, TI activities were indeed induced upon *S*. *littoralis* feeding. Although TI of plants are among the best studied proteins in plant biochemistry and biology, there has been no prior studies done on carnivorous plants that showed accumulation of TI upon herbivory. The role of protease inhibitors in plant defense against insects was demonstrated first when Green and Ryan [55] showed the induction of TIs in leaves of potato in response to wounding and insect feeding. Protease inhibitors are polypeptides and proteins that bind to proteolytic enzymes and prevent them from catalyzing [56]. Most of these inhibitors are unique to serine class proteinases, usually found in insects as the main food protein digestive enzymes [57]. Enzyme assays for proteinase inhibitors from our present study showed inhibition of the serine protease trypsin in extracts from herbivory-infested leaves, whereas non-infested leaf extracts showed no significant trypsin activity inhibition. Because TI accumulation occurred upon herbivory and is absent in unstressed leaves, it indicates that in *Nepenthes* TI are part of the inducible defense response.

In their natural environment, plants come across several pests and pathogens. Plant’s defense against such a threat involves either a fast consolidation of pre-existing physical and chemical barriers and/or the synthesis of many defensive substances through the induction of gene expression [29, 58]. In this study, herbivore feeding on *N*. *x ventrata* leaves enhanced a defense response in the form of plumbagin with a 2 to 3-fold increased endogenous level compared to untreated leaves (Fig 4*B*). Conspicuously, plumbagin was induced only upon long-term feeding and not by treatment of any phytohormones, not even jasmonates (Fig 7). In contrast, all phytohormones induced TI activity indicating that the spray approach was successful (Fig 8*B*). On one hand, the absence of phytohormone inducibility was surprising as jasmonates are rapidly and highly upregulated suggesting that JA-dependent defense reactions and compounds are induced; obviously, in *Nepenthes* various inducible defensive mechanisms remain to be detected. On the other hand, the kinetics of jasmonate or plumbagin accumulation also does not match. It would be worth to elucidate which signals and processes are involved in plumbagin induction upon herbivory; for example, a role for signaling compounds such as reactive oxygen species is conceivable. Although the accumulation of plumbagin in carnivorous plants has been mentioned in both *in vivo* and *in vitro* studies using polysaccharide elicitors [19, 59, 60], also in those cases little is known on the exact molecular mechanism governing the elicitor-induced production and accumulation of this phenolic compound. However, the fact that plumbagin is only slightly induced upon herbivory and all tested *Nepenthes* tissues have a constitutive level [22, 23] classifies this NQ as phytoanticipin rather than a phytoalexin, maybe comparable with glucosinolates in Brassicaceae [61].

## Conclusion

In this present study, chemical defenses upon herbivore damage in leaves of the carnivorous plant *N*. *x ventrata* were identified: the constitutive presence of the NQ plumbagin and the induced trypsin protease inhibitor activities. The latter very likely supports the effect of plumbagin on growth inhibition in *S*. *littoralis* larvae. Due to the constitutive presence of plumbagin, *Nepenthes* seems to be permanently well protected. Nevertheless, inducible defense responses such as the TI activity can support the first line of defense represented by the phytoanticipins. Strikingly, only TI induction is jasmonate-mediated which raises the question of how the synthesis and accumulation of plumbagin is regulated. From the data obtained so far, it is difficult to infer which signaling molecule may actually be involved in the accumulation of plumbagin in the long term. It will be furthermore interesting to examine if and what kind of other defense responses occur in *Nepenthes* and how those are regulated.

## Supporting information

S1 Fig Setup of *Spodoptera littoralis* feeding experiments on *Nepenthes x ventrata* leaves. (A) *N*. *x ventrata* (natural hybrid of *N*. *alata x N*. *ventricosa)* plant. (B) *S*. *littoralis* larvae together with the leaf were covered with a PET bag to prevent escaping of the larvae. For control, larvae were placed close to the plant but fed on artificial diet.
(PPTX)

S2 Fig Choice experiment; *Spodoptera littoralis* feeding on different tissues. (A) Setup in a 12 x 12 cm petri dish containing pieces of *Nepenthes x ventrata* pitcher (red) and leaf (green) tissue on a moist filter paper; an additional filter control was placed as well. (B) Photos of leaf and pitcher pieces with visiting larvae taken at the indicated time points.
(PPTX)

S3 Fig Jasmonic acid-isoleucine conjugate (JA-Ile) degradation products in *Nepenthes x ventrata* leaves upon *Spodoptera littoralis* larvae feeding. (A) OH-JA-Ile content after short term and (B) long term feeding. (C) COOH-JA-Ile content after short term and (D) long term feeding. Mean (± SE), *n* = 10; unpaired t-test with Welch correction; *** *p* < 0.001; ** *p* < 0.01.
(PPTX)

S1 Table Details of phytohormones and plumbagin analyses by LC-MS/MS. HPLC 1260 (Agilent Technologies)-QTRAP6500 (SCIEX)] in negative ionization mode.
(PPTX)

S1 File Data. (XLSX)

## References

[1] Darwin CR.1875. *Insectivorous Plants*. London, UK, John Murray.

[2] Juniper BE, Robins RJ, Joel DM (eds) 1989. *The Carnivorous Plants*. London, UK, Academic Press.

[3] Adamec L. 1997. Mineral nutrition of carnivorous plants: a review. *Bot*. *Rev*. 63, 273–299. doi: 10.1007/BF02857953

[4] Albert VA, Williams SE, Chase MW. 1992. Carnivorous plants: phylogeny and structural evolution. *Science* 257, 1491–1495. doi: 10.1126/science.1523408 1523408

[5] Ellison AM, Gotelli NJ. 2009. Energetics and the evolution of carnivorous plants—Darwin’s “most wonderful plants in the world.” *J*. *Exp*. *Bot*. 60, 19–42. doi: 10.1093/jxb/ern179 19213724

[6] Chase MW, Christenhusz MJM. Sanders D, Fay MF. 2009. Murderous plants: Victorian gothic, Darwin and modern insights into vegetable carnivory. *Bot*. *J*. *Linn*. *Soc*. 161, 329–356. doi: 10.1111/j.1095-8339.2009.01014.x

[7] Ellison AM, Adamec L, eds. 2018. *Carnivorous Plants*: *Physiology*, *Ecology*, *and Evolution*. Oxford, UK, Oxford University Press. doi: 10.1093/oso/9780198779841.001.0001

[8] Hedrich R, Fukushima K. 2021. On the origin of carnivory: Molecular physiology and evolution of plants on an animal diet. *Annu*. *Rev*. *Plant Biol*. 72, 1. doi: 10.1146/annurev-arplant-071720-111039 33434053

[9] Miguel S, Hehn A, Bourgaud F. 2018. *Nepenthes*: State of the art of an inspiring plant for biotechnologists. *J*. *Biotechnol*. 265, 109–115. doi: 10.1016/j.jbiotec.2017.11.014 29191666

[10] Legendre G, Darnowski DW. 2018. Biotechnology with carnivorous plants. In *Carnivorous plants*: *physiology*, *ecology*, *and evolution* (eds Ellison AM, Adamec L), pp. 270–282. Oxford, UK, Oxford University Press. doi: 10.1093/oso/9780198779841.003.0020

[11] Chi VV. 2012. *Dictionary of Vietnamese Medicinal Plants*, *Volume* 2. Hanoi, Vietnam, Publishing House Medicine.

[12] Likhitwitayawuid K, Kaewamatawong R, Ruangrungsi N, Krungkrai J. 1998. Antimalarial naphthoquinones from *Nepenthes thorelii*. *Planta Med*. 64, 237–241. doi: 10.1055/s-2006-957417 9581522

[13] D’Amoto P. 1998. *The Savage Garden*. Berkeley, CA, USA, Ten Speed Press.

[14] Wiard C, Morgana S, Khalifah S, Mahan M, Ismael S, Buckle M, et al. 2004. Antimicrobial screening of plants used for traditional medicine in the state of Perak, Peninsula Malaysia. *Fitoterapia* 75, 68–73. doi: 10.1016/j.fitote.2003.07.013 14693223

[15] Rey M, Yang M, Lee L, Zhang Y, Sheff JG, Sensen CW, et al. 2016. Addressing proteolytic efficiency in enzymatic degradation therapy for celiac disease. *Sci*. *Rep*. 6, 30980. doi: 10.1038/srep30980 27481162 PMC4969619

[16] Tang J-Y, Peng S-Y, Cheng Y-B, Wang C, Farooqi AA, Yu T-J, et al. 2019. Ethyl acetat extract of Nepenthes adrianii x clipeata induces antiproliferation, apoptosis, and DNA damage against oral cancer cells through oxidative stress. *Environ*. *Toxcol*. 34, 891–901. doi: 10.1002/tox.22748 31157515

[17] Schlauer J, Nerz J, Rischer H. 2005. Carnivorous plant chemistry. *Acta Bot*. *Gall*. 152, 187–195. doi: 10.1080/12538078.2005.10515469

[18] Hatcher CR, Ryves DB, Millett J. 2020. The function of secondary metabolites in plant carnivory. *Ann*. *Bot*. 125, 399–411. doi: 10.1093/aob/mcz191 31760424 PMC7061172

[19] Eilenberg H, Pnini-Cohen S, Rahamim Y, Sionov E, Segal E, Carmel S, et al. 2010. Induced production of antifungal naphthoquinones in the pitchers of the carnivorous plant *Nepenthes khasiana*. *J*. *Exp*. *Bot*. 61, 911–922. doi: 10.1093/jxb/erp359 20018905 PMC2814117

[20] Buch F, Rott M, Rottloff S, Paetz C, Hilke I, Raessler M, et al. 2013. Secreted pitfall-trap fluid of carnivorous *Nepenthe*s plants is unsuitable for microbial growth. *Ann*. *Bot*.111, 375–383. doi: 10.1093/aob/mcs287 23264234 PMC3579442

[21] Mithöfer A. 2011. Carnivorous pitcher plants: Insights in an old topic. *Phytochemistry* 72, 1678–1682. doi: 10.1016/j.phytochem.2010.11.024 21185041

[22] Raj G, Kurup R, Hussain AA, Baby S. 2011. Distribution of naphthoquinones, plumbagin, droserone, and 5-O-methyl droserone in chitin-induced and uninduced *Nepenthes khasiana*: Molecular events in prey capture. *J*. *Exp*. *Bot*. 62, 5429–5436. doi: 10.1093/jxb/err219 21862483

[23] Rahman-Soad A, Dávila-Lara A, Paetz C, Mithöfer A. 2021. Plumbagin, a potent naphthoquinone from *Nepenthes* plants with growth inhibiting and larvicidal activities. *Molecules* 26, 825. doi: 10.3390/molecules26040825 33562562 PMC7915728

[24] Dávila-Lara A, Rodríguez-López CE, O’Connor SE, Mithöfer A. 2020. Metabolomics analysis reveals tissue-specific metabolite compositions in leaf blade and traps of carnivorous *Nepenthes* plants. *Int*. *J*. *Mol*. *Sci*. 21, 4376. doi: 10.3390/ijms21124376 32575527 PMC7352528

[25] Carmickle RN, Horner JD. 2019. Impact of the specialist herbivore *Exyra semicrocea* on the carnivorous plant *Sarracenia alata*: A field experiment testing the effects of tissue loss and diminished prey capture on plant growth. *Plant Ecol*. 220, 553–561. doi: 10.1007/s11258-019-00935-y

[26] Lamb T, Kalies EL. 2020. An overview of lepidopteran herbivory on North American pitcher plants (*Sarracenia*), with a novel observation of feeding on *Sarracenia flava*. *J*. *Lepid*. *Soc*. 2020, 74, 193–197. doi: 10.18473/lepi.74i3.a7

[27] Merbach MA, Zizka G, Fiala B, Merbach D, Booth WE, Maschwitz U. 2007. Why a carnivorous plant cooperates with an ant–selective defense against pitcher-nutritional mutualism in a pitcher plant destroying weevils in the myrmecophytic pitcher plant *Nepenthes bicalcarata* Hook. F. *Ecotropica* 13, 45–56.

[28] Gilbert KJ, Nitta JH, Talavera G, Pierce NE. 2018. Keeping an eye on coloration: Ecological correlates of the evolution of pitcher traits in the genus *Nepenthes* (Caryophyllales). *Biol*. *J*. *Linn*. *Soc*.123, 321–337. doi: 10.1093/biolinnean/blx142

[29] Mithöfer A, Boland W. 2012. Plant defense against herbivores: Chemical aspects. *Annu*. *Rev*. *Plant Biol*. 63, 431–450. doi: 10.1146/annurev-arplant-042110-103854 22404468

[30] Pavlovič A, Mithöfer A. 2019. Jasmonate signalling in carnivorous plants: Copycat of plant defence mechanisms. J. Exp. Bot. 70, 3379–3389. doi: 10.1093/jxb/erz188 31120525

[31] Vadassery J, Reichelt M, Hause B, Gershenzon J, Boland W, Mithöfer A. 2012. CML42-mediated calcium signaling co-ordinates responses to *Spodoptera* herbivory and abiotic stresses in Arabidopsis. *Plant Physiol*. 159, 1159–1175. doi: 10.1104/pp.112.198150 22570470 PMC3387702

[32] Heyer M, Reichelt M, Mithöfer A. 2018. A holistic approach to analyze systemic jasmonate accumulation in individual leaves of *Arabidopsis* rosettes upon wounding. *Front*. *Plant Sci*. 9, 1569. doi: 10.3389/fpls.2018.01569 30425725 PMC6218591

[33] Kuhar K, Kansal R, Subrahmanyam B, Koundal KR, Miglani K, Gupta VK. 2013. A Bowman-Birk protease inhibitor with antifeedant and antifungal activity from *Dolichos biflorus*. *Acta Physiol*. *Plant* 35, 1887–1903. doi: 10.1007/s11738-013-1227-8

[34] Devi SP, Kumaria S, Rao SR, Tandon P. 2016. Carnivorous Plants as a Source of Potent Bioactive Compound: Naphthoquinones. *Trop*. *Plant Biol*. 9, 267–279. doi: 10.1007/s12042-016-9177-0

[35] Fleischmann A, Schlauer J, Smith S, Givnish TJ. 2018. Evolution of carnivory in angiosperms. In *Carnivorous plants*: *physiology*, *ecology*, *and evolution* (eds Ellison AM, Adamec L), pp. 22–42. Oxford, UK, Oxford University Press. doi: 10.1093/oso/9780198779841.003.0003

[36] Wolfson JL. 1988. Bioassay techniques. *J*. *Chem*. *Ecol*. 14, 1951–1963. doi: 10.1007/BF01013488 24277105

[37] Reese J, Field MD. 1986. Defense against insect attack in susceptible plants: black cutworm (Lepidoptera: Noctuidae) growth on corn seedlings and artificial diet. *Ann*. *Entomol*. *Soc*. *Am*. 79, 372–376. doi: 10.1093/aesa/79.2.372

[38] Sreelatha T, Hymavathi A, Babu KS, Murthy JM, Pathipati UR, Rao JM. 2009. Synthesis and insect antifeedant activity of plumbagin derivatives with the amino acid moiety. *Agric*. *Food Chem*. 57, 6090–6094. doi: 10.1021/jf901760h 19530696

[39] Tokunaga T, Takada N, Ueda M. 2004. Mechanism of antifeedant activity of plumbagin, a compound concerning the chemical defense in carnivorous plant. *Tetrahedron Lett*. 45, 7115–7119. doi: 10.1016/j.tetlet.2004.07.094

[40] Akhtar Y, Isman MB, Niehaus LA, Lee C-H, Lee H-S. 2012. Antifeedant and toxic effects of naturally occurring and synthetic quinones to the cabbage looper, *Trichoplusia ni*. *Crop Prot*. 31, 8–14. doi: 10.1016/j.cropro.2011.09.009

[41] Cook SM, Khan ZR, Pickett JA. 2007. The use of push-pull strategies in integrated pest management. *Annu*. *Rev*. *Entomol*. 52, 375–400. doi: 10.1146/annurev.ento.52.110405.091407 16968206

[42] Bennett KF, Ellison AM. 2009. Nectar, not colour, may lure insects to their death. *Biol*. *Lett*. 5, 469–472. doi: 10.1098/rsbl.2009.0161 19429649 PMC2781919

[43] Maffei ME, Mithöfer A, Boland W. 2007. Before gene expression: early events in plant–insect interaction. *Trends Plant Sci*. 12, 310–316. doi: 10.1016/j.tplants.2007.06.001 17596996

[44] Ueda M, Tokunaga T, Okada M, Nakamura Y, Takada N, Suzuki R, et al. 2010. Trap-closing chemical factors of the Venus Flytrap (*Dionaea muscipula* Ellis). *Chembiochem*. 11, 2378–2383. doi: 10.1002/cbic.201000392 20963745

[45] Escalante-Pérez M, Krol E, Stange A, Geiger D, Al-Rasheid KA, Hause B, et al. 2011. A special pair of phytohormones controls excitability, slow closure, and external stomach formation in the Venus flytrap. *Proc*. *Natl*. *Acad*. *Sci*. USA 108, 15492–15497. doi: 10.1073/pnas.1112535108 21896747 PMC3174645

[46] Libiaková M, Floková K, Novák O, Slováková L, Pavlovič A. 2014. Abundance of cysteine endopeptidase dionain in digestive fluid of Venus, flytrap (*Dionaea muscipula* Ellis) is regulated by different stimuli from prey through jasmonates. PLoS One 9, e104424. doi: 10.1371/journal.pone.0104424 25153528 PMC4143254

[47] Bemm F, Becker D, Larisch C, Kreuzer I, Escalante-Perez M, Schulze WX, et al. 2016. Venus flytrap carnivorous lifestyle builds on herbivore defense strategies. *Genome Res*. 26, 812–825. doi: 10.1101/gr.202200.115 27197216 PMC4889972

[48] Nakamura Y, Reichelt M, Mayer VE, Mithöfer A. 2013. Jasmonates trigger prey-induced formation of ‘outer stomach’ in carnivorous sundew plants. *Proc*. *Royal Soc*. *B*, 20130228. doi: 10.1098/rspb.2013.0228 23516244 PMC3619512

[49] Yilamujiang A, Reichelt M, Mithöfer A. 2016. Slow food: Insect prey and chitin induce phytohormone accumulation and gene expression in carnivorous *Nepenthes* plants. *Ann*. *Bot*. 118, 369–375. doi: 10.1093/aob/mcw110 27325901 PMC4970371

[50] Mithöfer A, Reichelt M, Nakamura Y. 2014. Wound and insect-induced jasmonate accumulation in carnivorous *Drosera capensis*: Two sides of the same coin. *Plant Biol*. 16, 982–987. doi: 10.1111/plb.12148 24499476

[51] Enyedi AJ, Yalpani N, Silverman P, Raskin I. 1992. Localization, conjugation, and function of salicylic acid in tobacco during the hypersensitive reaction to tobacco mosaic virus. *Proc*. *Natl*. *Acad*. *Sci*. *USA* 89, 2480–2484. doi: 10.1073/pnas.89.6.2480 1549613 PMC48682

[52] Malamy J, Carr J, Klessig D, Raskin I. 1990. Salicylic acid: A likely endogenous signal in the resistance response of tobacco to viral infection. *Science* 250, 1002–1004. doi: 10.1126/science.250.4983.1002 17746925

[53] Tuteja N. 2007. Abscisic acid and abiotic stress signaling. *Plant Signal*. *Behav*. 2, 135–138. doi: 10.4161/psb.2.3.4156 19516981 PMC2634038

[54] Weldegergis BT, Zhu F, Poelman EH, Dicke M. 2015. Drought stress affects plant metabolites and herbivore preference but not host location by its parasitoids. *Oecologia* 177, 701–713. doi: 10.1007/s00442-014-3129-x 25370387

[55] Green TR, Ryan CA. 1972. Wound-induced proteinase inhibitor in plant leaves: A possible defense mechanism against Insects. *Science* 175, 776–777. doi: 10.1126/science.175.4023.776 17836138

[56] Clemente M, Corigliano MG, Pariani SA, Sánchez-López EF, Sander VA, Ramos-Duarte VA. 2019. Plant serine protease inhibitors: Biotechnology application in agriculture and molecular farming. *Int*. *J*. *Mol*. *Sci*. 20, 1345. doi: 10.3390/ijms20061345 30884891 PMC6471620

[57] Jamal F, Pandey PK, Singh D, Khan MY. 2013. Serine protease inhibitors in plants: nature’s arsenal crafted for insect predators. *Phytochem*. *Rev*. 12, 1–34. doi: 10.1007/s11101-012-9231-y

[58] Maffei ME, Arimura G, Mithöfer A. 2012. Natural elicitors, effectors and modulators of plant responses. *Nat*. *Prod*. *Rep*. 29, 1288–303. doi: 10.1039/c2np20053h 22918379

[59] Marczak Ł, Kawiak A, Łojkowska E, Stobiecki M. 2005. Secondary metabolites in in vitro cultured plants of the genus *Drosera*. *Phytochem*. *Anal*. 16, 143–149. doi: 10.1002/pca.833 15997845

[60] Nahálka J, Nahálková J, Gemeiner P, Blanárik P. 1998. Elicitation of plumbagin by chitin and its release into the medium in *Drosophyllum lusitanicum* Link. suspension cultures. *Biotechnol*. *Lett*. 20, 841–845. doi: 10.1023/A:1005307408135

[61] Halkier BA, Gershenzon J. 2006. Biology and biochemistry of glucosinolates. *Annu*. *Rev*. *Plant Biol*. 57, 303–333. doi: 10.1146/annurev.arplant.57.032905.105228 16669764

