# Supplementary material for: Carnivorous Nepenthes x ventrata plants use a naphthoquinone as phytoanticipin against herbivory
Source: PLoS One. 2021 Oct 22;16(10):e0258235. doi: 10.1371/journal.pone.0258235 (PMC8535358; doi:10.1371/journal.pone.0258235)
Supplement: S1 Fig — (A) N. x ventrata (natural hybrid of N. alata x N. ventricosa) plant. (B) S. littoralis larvae together with the leaf were covered with a PET bag to prevent escaping of the larvae. For control, larvae were placed close to the plant but fed on artificial diet. (PPTX) [file pone.0258235.s001.pptx]

## Slide 1
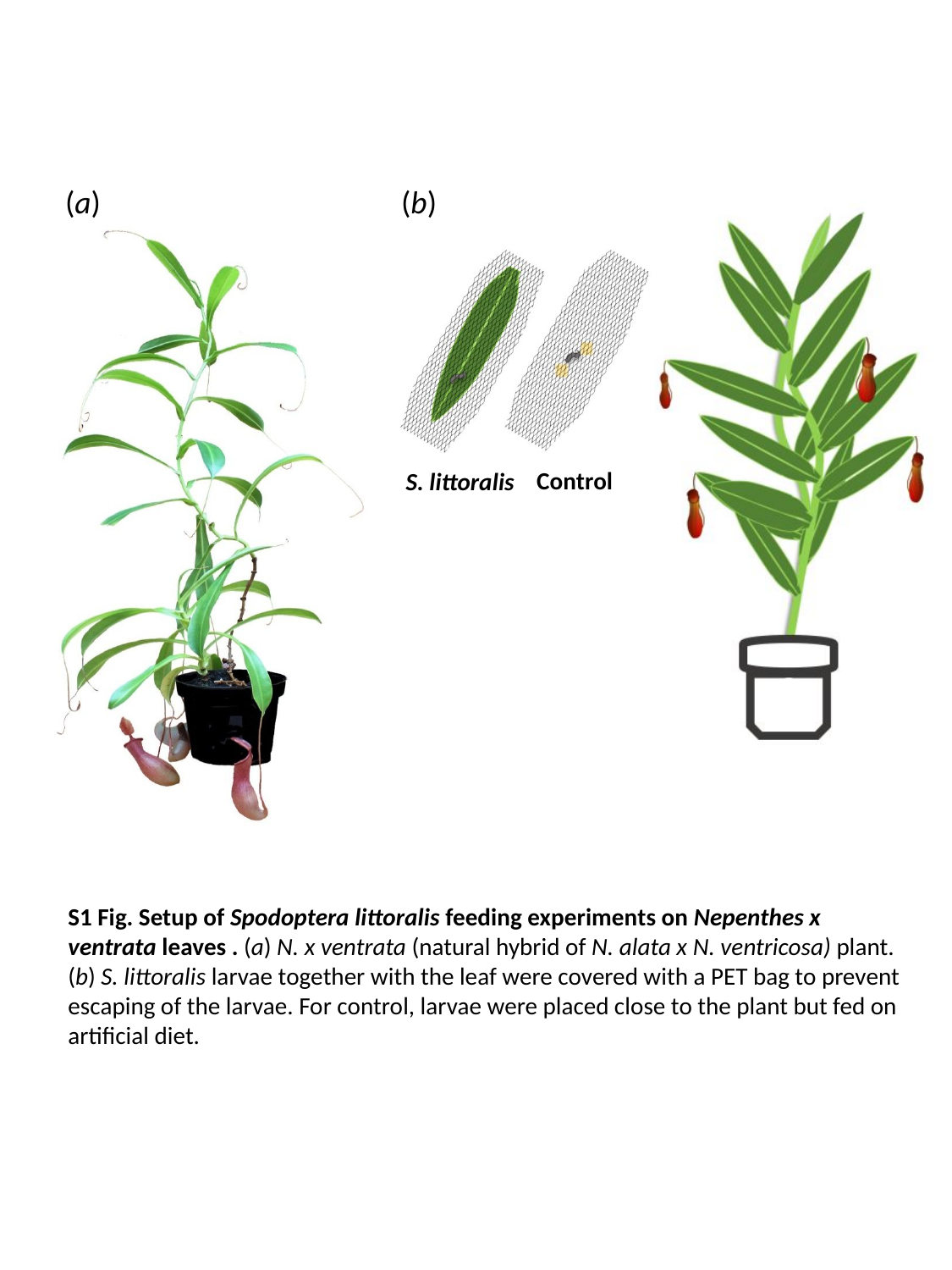

(a)
(b)
Control
S. littoralis
S1 Fig. Setup of Spodoptera littoralis feeding experiments on Nepenthes x ventrata leaves . (a) N. x ventrata (natural hybrid of N. alata x N. ventricosa) plant. (b) S. littoralis larvae together with the leaf were covered with a PET bag to prevent escaping of the larvae. For control, larvae were placed close to the plant but fed on artificial diet.
