# Supplementary material for: Carnivorous Nepenthes x ventrata plants use a naphthoquinone as phytoanticipin against herbivory
Source: PLoS One. 2021 Oct 22;16(10):e0258235. doi: 10.1371/journal.pone.0258235 (PMC8535358; doi:10.1371/journal.pone.0258235)
Supplement: S2 Fig — (A) Setup in a 12 x 12 cm petri dish containing pieces of Nepenthes x ventrata pitcher (red) and leaf (green) tissue on a moist filter paper; an additional filter control was placed as well. (B) Photos of leaf and pitcher pieces with visiting larvae taken at the indicated time points. (PPTX) [file pone.0258235.s002.pptx]

## Slide 1
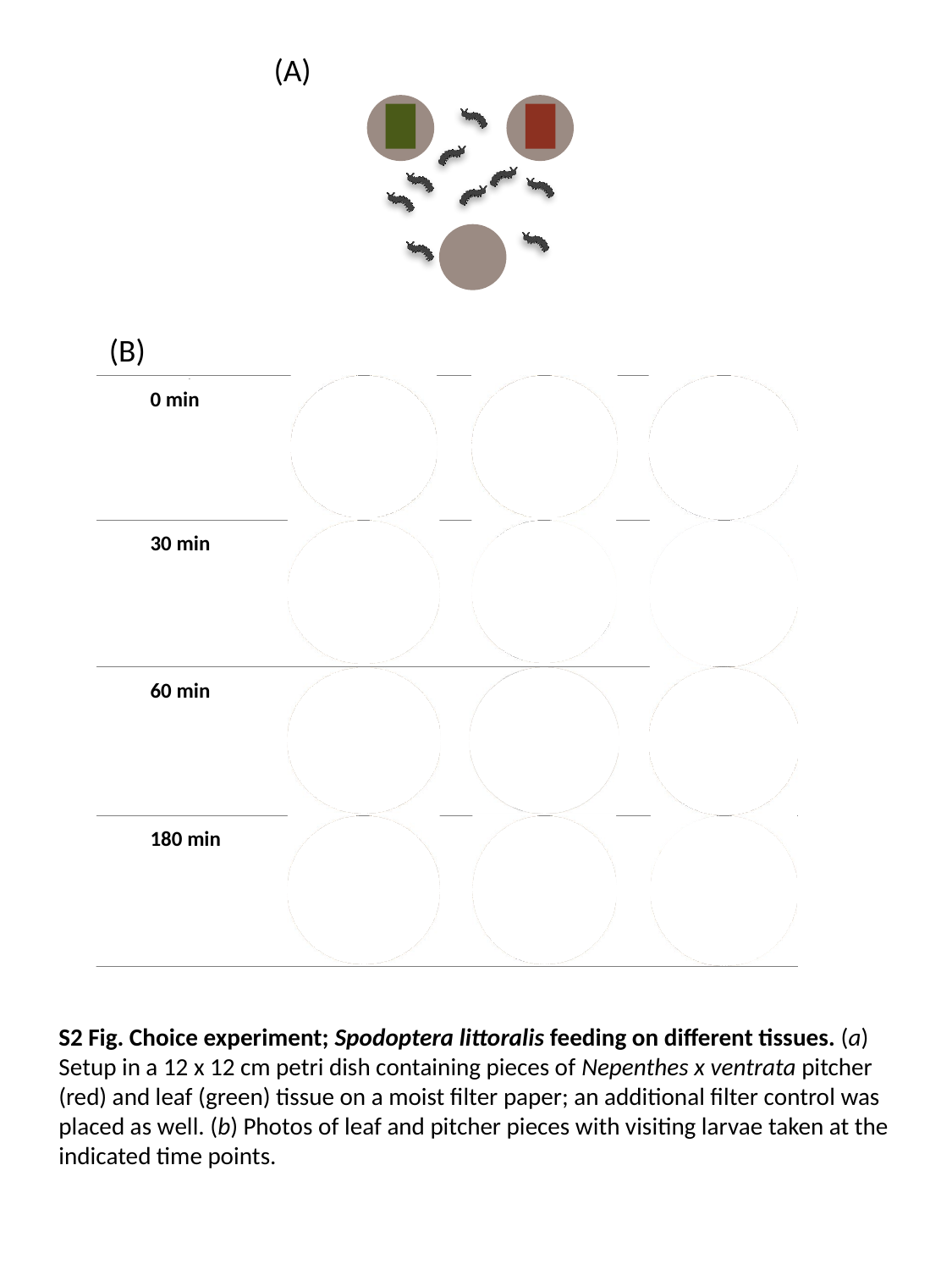

(A)
(B)
0 min
30 min
60 min
180 min
S2 Fig. Choice experiment; Spodoptera littoralis feeding on different tissues. (a) Setup in a 12 x 12 cm petri dish containing pieces of Nepenthes x ventrata pitcher (red) and leaf (green) tissue on a moist filter paper; an additional filter control was placed as well. (b) Photos of leaf and pitcher pieces with visiting larvae taken at the indicated time points.
