# Supplementary material for: Carnivorous Nepenthes x ventrata plants use a naphthoquinone as phytoanticipin against herbivory
Source: PLoS One. 2021 Oct 22;16(10):e0258235. doi: 10.1371/journal.pone.0258235 (PMC8535358; doi:10.1371/journal.pone.0258235)
Supplement: S3 Fig — (A) OH-JA-Ile content after short term and (B) long term feeding. (C) COOH-JA-Ile content after short term and (D) long term feeding. Mean (± SE), n = 10; unpaired t-test with Welch correction; *** p < 0.001; ** p < 0.01. (PPTX) [file pone.0258235.s003.pptx]

## Slide 1
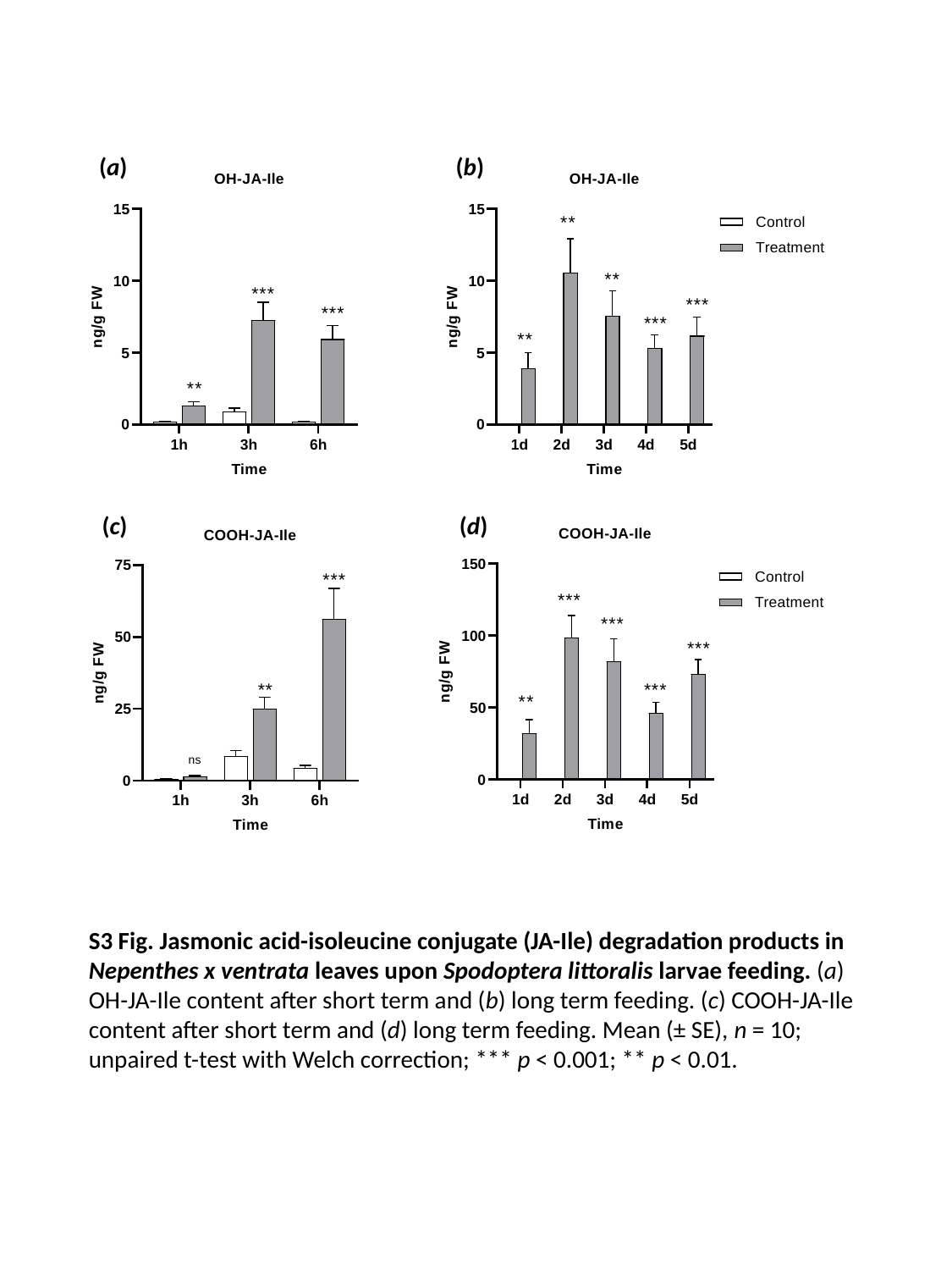

(a)
(b)
(d)
(c)
S3 Fig. Jasmonic acid-isoleucine conjugate (JA-Ile) degradation products in Nepenthes x ventrata leaves upon Spodoptera littoralis larvae feeding. (a) OH-JA-Ile content after short term and (b) long term feeding. (c) COOH-JA-Ile content after short term and (d) long term feeding. Mean (± SE), n = 10; unpaired t-test with Welch correction; *** p < 0.001; ** p < 0.01.
