# Supplementary material for: Carnivorous Nepenthes x ventrata plants use a naphthoquinone as phytoanticipin against herbivory
Source: PLoS One. 2021 Oct 22;16(10):e0258235. doi: 10.1371/journal.pone.0258235 (PMC8535358; doi:10.1371/journal.pone.0258235)
Supplement: S1 Table — HPLC 1260 (Agilent Technologies)-QTRAP6500 (SCIEX)] in negative ionization mode. (PPTX) [file pone.0258235.s004.pptx]

## Slide 1
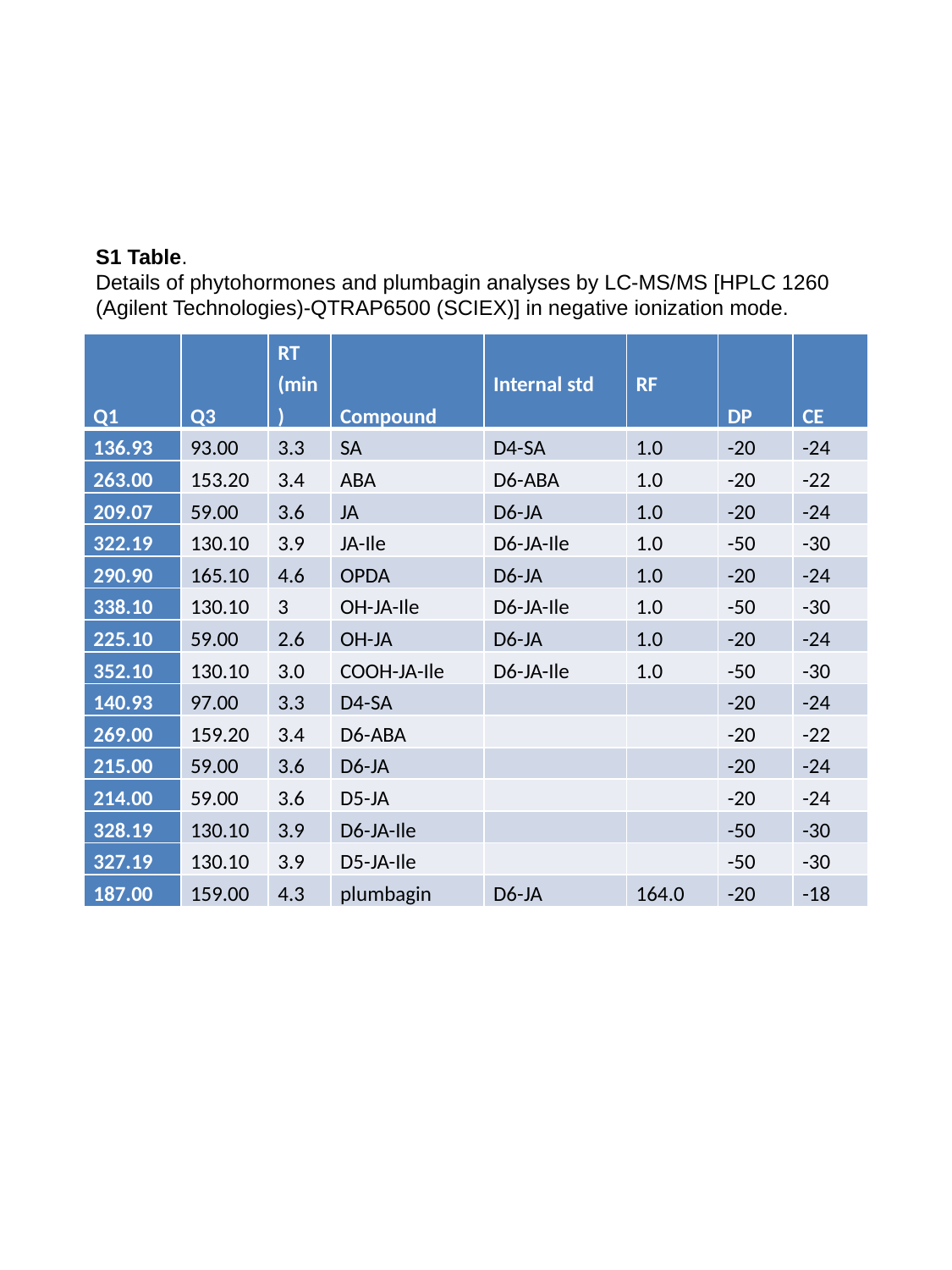

S1 Table.
Details of phytohormones and plumbagin analyses by LC-MS/MS [HPLC 1260
(Agilent Technologies)-QTRAP6500 (SCIEX)] in negative ionization mode.
| Q1 | Q3 | RT (min) | Compound | Internal std | RF | DP | CE |
| --- | --- | --- | --- | --- | --- | --- | --- |
| 136.93 | 93.00 | 3.3 | SA | D4-SA | 1.0 | -20 | -24 |
| 263.00 | 153.20 | 3.4 | ABA | D6-ABA | 1.0 | -20 | -22 |
| 209.07 | 59.00 | 3.6 | JA | D6-JA | 1.0 | -20 | -24 |
| 322.19 | 130.10 | 3.9 | JA-Ile | D6-JA-Ile | 1.0 | -50 | -30 |
| 290.90 | 165.10 | 4.6 | OPDA | D6-JA | 1.0 | -20 | -24 |
| 338.10 | 130.10 | 3 | OH-JA-Ile | D6-JA-Ile | 1.0 | -50 | -30 |
| 225.10 | 59.00 | 2.6 | OH-JA | D6-JA | 1.0 | -20 | -24 |
| 352.10 | 130.10 | 3.0 | COOH-JA-Ile | D6-JA-Ile | 1.0 | -50 | -30 |
| 140.93 | 97.00 | 3.3 | D4-SA | | | -20 | -24 |
| 269.00 | 159.20 | 3.4 | D6-ABA | | | -20 | -22 |
| 215.00 | 59.00 | 3.6 | D6-JA | | | -20 | -24 |
| 214.00 | 59.00 | 3.6 | D5-JA | | | -20 | -24 |
| 328.19 | 130.10 | 3.9 | D6-JA-Ile | | | -50 | -30 |
| 327.19 | 130.10 | 3.9 | D5-JA-Ile | | | -50 | -30 |
| 187.00 | 159.00 | 4.3 | plumbagin | D6-JA | 164.0 | -20 | -18 |
